# Effect of remote ischemic preconditioning on postoperative cognitive dysfunction in adult patients with general anesthesia: a meta-analysis

**DOI:** 10.1186/s13019-024-02520-5

**Published:** 2024-02-06

**Authors:** Mengnan Han, Yanru Du, Jianli Li, Yi Li, Shuang Han, Chong Li

**Affiliations:** 1 Department of Anesthesiology, Hebei General Hospital, No 348 West Heping Road, Shijiazhuang, 050051 China; 2 Graduate Faculty, Hebei North University, Zhangjiakou, China

**Keywords:** General surgery, General anesthesia, Remote ischemic preconditioning, Postoperative complications, Cognitive dysfunction, Neuropsychological tests, Meta-analysis

## Abstract

**Background:**

Remote ischemic preconditioning (RIPC) is proven to have neuroprotective protective effects. Nevertheless, the impact of RIPC on postoperative cognitive dysfunction (POCD) in patients undergoing general anesthesia is controversial. This meta-analysis of randomized controlled trials (RCTs) aimed to assess the effect of RIPC on POCD in adults after general anesthesia.

**Methods:**

Relevant literature was obtained by searching Embase, PubMed, Web of Science, Cochrane Library, Wanfang, and China National Knowledge Infrastructure (CNKI) databases in July 2022. RCTs were included to assess the influences of RIPC on POCD in adults following general anesthesia. Two investigators independently performed literature screening, data extraction, and quality assessment based on the inclusion and exclusion criteria. The incidence of POCD, operation time, and hospital stay were analyzed by Review manager5.4 software.

**Results:**

Thirteen RCTs with 1122 participants were selected for this meta-analysis. Compared to the control group, RIPC decreased the incidence of POCD (OR = 0.50, 95% CI 0.31–0.82), as well as reduced the duration of hospitalization (MD = − 0.98, 95% CI − 1.69 to − 0.27), but did not prolong operative time (MD = − 2.65, 95% CI − 7.68 to 2.37).

**Conclusion:**

RIPC reduced the incidence of POCD in adult patients after general anesthesia and accelerated their discharge.

## Introduction

Postoperative cognitive dysfunction (POCD) is one of the central nervous system complications after general anesthesia surgery, especially in elderly patients. It referred to the decline of various cognitive functions from baseline, such as attention, fluency of language, and executive function [1]. The clinical manifestations of POCD were mainly characterized by changes in consciousness, disturbance of thinking, psychomotor speed, memory, sleep, and learning disabilities [2]. Studies have shown that the incidence of POCD one week after surgery varies from 26 to 53% depending on the type of surgery, anesthesia protocol, and assessment methods [3, 4]. POCD could lead to loss of speech, personality changes, and even permanent illnesses such as Alzheimer’, which might persist for weeks, months, or even longer after surgery [5, 6]. In the long run, it reduced the possibility of patients returning to independent living and burdened families and society [7, 8]. However, the exact pathophysiological mechanism of POCD remains unknown [9]. Studies showed that POCD was associated with inflammatory response [10, 11]. Specific drugs and interventions are currently unavailable to treat POCD, and the only solution seems to be prevention.

Remote ischemic preconditioning (RIPC) is an approach to protect against subsequent vital organ damage through nonlethal ischemic-reperfusion of distal ischemic-tolerant tissues. An animal experiment revealed that RIPC could protect the brain from damage during hypothermic circulatory arrest [12]. In a clinical study, RIPC appeared safe in patients because it increased the tolerance of tissue vulnerability in the brain, and no adverse effects were reported [13]. Furthermore, RIPC was safe, non-invasive, convenient, and feasible with significant clinical application value.

It’s worthwhile to note that RIPC has been shown to improve POCD in elderly patients undergoing colon surgery [14]. A recent meta-analysis, however, revealed that RIPC had no beneficial effect on POCD in adults after cardiac surgery [15]. Whether RIPC could improve POCD was debatable in previous studies. To date, no meta-analysis was performed on the influence of RIPC on POCD in adult patients after general anesthesia surgery. The purpose of our meta-analysis was to systematically and comprehensively summarize and discuss the impact of RIPC on POCD in adults following general anesthesia.

## Methods

### Search strategy

The research was designed and implemented according to the Preferred Reporting Items for Systematic Reviews and Meta-Analyses(PRISMA)statement [16]. Embase, PubMed, Web of Science, Cochrane Library, Wanfang, and CNKI databases were retrieved by two researchers to find relevant studies that only included human subjects without any language restrictions. Comprehensive search strategies were (1) "RIPC" OR "remote ischemic preconditioning" OR "distal ischemic preconditioning“, (2) "postoperative cognitive dysfunction" OR "Cognitive Complication" OR “POCD”, (3) "Randomized controlled trial" OR “randomized” OR “random”. References to relevant articles were also screened as a supplement. The last database retrieval occurred on July 31, 2022.

### Inclusion and exclusion criteria

The literature was eligible based on the following inclusion criteria: (1) patients in adults undergoing general anesthesia without limiting the type of surgery; (2) randomized controlled trials in humans (RCTs); (3) no neurological or psychiatric history, and Preoperative Mini-Mental State Examination (MMSE) score ≥ 24; (4) Intervention: RIPC of upper or lower limbs in the trial group; The influence of RIPC on POCD was discussed, and the incidence of POCD, surgery time and hospitalization time were reported.

The exclusion criteria for articles were as follows: (1) case reports, animal experiments, systematic reviews, meeting materials, and duplicative publications; (2) cognitive function was not assessed before surgery; (3) failure to collect complete clinical data or obtain the full text.

### Quality assessment

Two reviewers independently evaluated the risk of bias in the eligible literature using the Cochrane risk of bias tool [17]. According to seven criteria, including random sequence generation, allocation concealment, blinding of participants and personnel, blinding of outcome assessment, incomplete outcome data, selective reporting, and other biases, the risk of bias for each article was assessed as unclear, low, and high. Disputes between reviewers were discussed with a third reviewer when necessary and resolved by consensus.

### Data extraction and outcomes

Two researchers collected all the data of the studies from the screened literature using a standardized data sheet. If disagreements existed, the issue was resolved through discussion. The relevant information extracted included the year of publication, authors, country, blinding, the number of patients, male and mean age, RIPC method, diagnostic criteria for POCD, type of operation, and anesthesia protocol. The incidence of POCD was the main result of this meta-analysis. Surgery time and the total hospital stay were secondary outcomes.

### Statistical analysis

Review Manager 5.4 software was applied to perform statistical analysis. We calculated odds ratios (ORs) and their corresponding 95% confidence intervals (CIs) using a random effects model to represent effect sizes for dichotomous outcomes. Mean differences (MDs) and corresponding 95% CIs were used to analyze continuous outcomes. We used the I-square (I^2^) test to evaluate the heterogeneity of the included literature. If the articles showed high heterogeneity (*P* < 0.1 or I^2^ ≥ 50.0%), a random effects model was employed, and we performed a further sensitivity analysis to identify potential causes of heterogeneity.

## Results

### Study selection

**Fig. 1:**
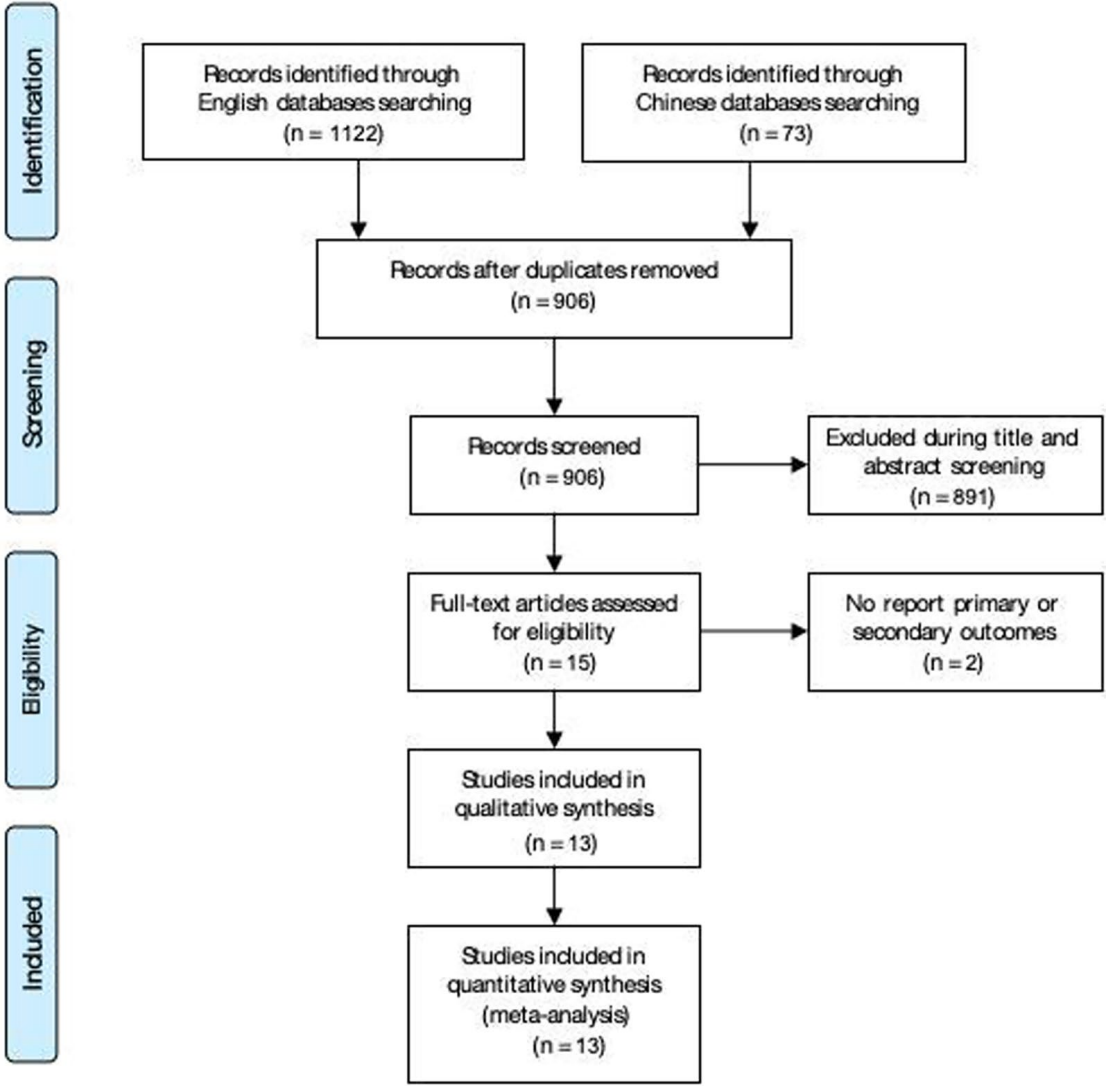
Flow diagram of the trials screening procedures

**Fig. 2:**
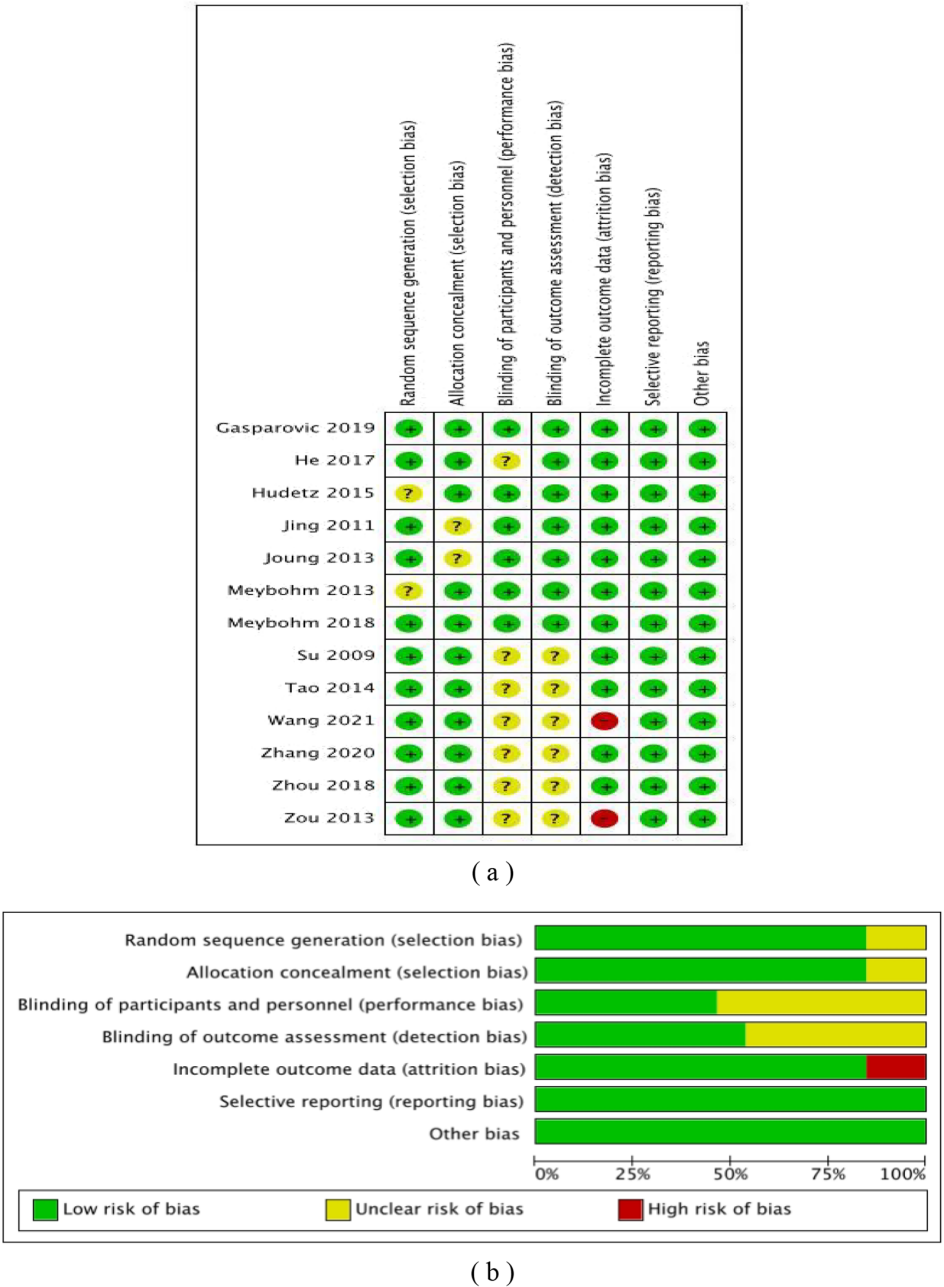
Risk bias assessment of Cochrane. **a** Risk of bias summary. A summary table of review authors’ judgments for each risk of bias item for each study; **b** Risk of bias graph. A plot of the distribution of review authors’ assessments across studies for each risk of bias item. Note: “+” represents low risk; “?” represents unclear risk; “−” represents high risk

Database retrieval, articles review, and selection process were displayed in Fig. 1. In short, we initially identified 1195 potentially relevant articles. After removing duplicates, 906 unique references were selected. Eight hundred ninety-one articles were excluded by reading abstracts and titles. Fifteen papers were available for inclusion. Then, we removed two full-text articles for lack of vital results. In the end, we included 13 randomized controlled trials in our meta-analysis. The Cochrane risk of bias assessment results were shown in Fig. 2.

### Study characteristics

The characteristics of 13 articles were displayed in Table 1. All researches were RCTs involving 1122 patients, which were published from 2009 to 2021 and performed in China [18–24], Korea [25], Germany [26, 27], and the USA [28–30]. General intravenous anesthesia was used in all clinical experiments. In 12 research, the RIPC protocol was applied after induction of anesthesia, while in 1 study, it was performed before anesthesia induction. RIPC was performed by Ischemia of the upper or lower limbs for 3–4 cycles (the pressure of the cuff was pressurized to 200 mmHg or 35 Kpa for five min) and then deflated the cuff for five min. In this meta-analysis, POCD was identified within one week after surgery. However, the neuropsychological tests used to assess cognitive dysfunction were different. Specifically, POCD was defined by 1-SD in 3 studies, MoCA in 3 studies, Z score in 1 study, MMSE in 4 studies,1 study used both 1-SD and Z score, and the other used both MMSE and MoCA. Moreover, the types of procedures included in the RCTs were shown below: 8 were cardiac operations, 2 were liver resection, 1 was colon surgery, 1 was vascular revascularization for Moyamoya disease, and 1 literature included 7 types of surgical operations.

**Table 1:** Characteristics of the included RCTs

Study	County	Design	No. of patients	Mean age years	Male %	Anesthesia regimen	Type of surgery	Protocols of RIPC	Control	Diagnosis of outcomes
Hudetz 2015	USA	R, DB	30	65.5	100	Midazolam, fentanyl, rocuronium, etomidate, isoflurane;	On-pump heart surgery	UL, 200mmHg, 5min × 4, after anesthesia induction and before CPB	Uninflated cuff	1-SD
Gasparovic 2019	USA	R, DB	66	62	82	Midazolam, sufentanil, rocuronium, sevoflurane;	On-pump CABG	UL, 200mmHg, 5min × 3, after anesthesia induction and before CPB	Uninflated cuff	MoCA
Meybohm 2018	Germany	R, DB	273	NR	NR	Intravenous anesthesia with no volatile anesthetic agents	On-pump heart surgery	UL, 200mmHg, 5min × 4, after anaesthesia induction and before CPB	Uninflated cuff	1-SD and Z score
Meybohm 2013	Germany	R, DB	180	69	81.2	Propofol, rocuronium, sufentanil;	On-pump heart surgery	UL, 200mmHg, 5min × 4, after anesthesia induction and before CPB	Uninflated cuff	1-SD
Joung 2013	Korea	R, DB	70	60	81.4	Etomidate, propofol, rocuronium, remifentanil;	Off-pump CABG	UL, 200mmHg, 5min × 4, before coronary artery anastomosis	Uninflated cuff	1-SD
He 2017	China	R, DB	90	68	13.3	Midazolam, etomidate, sufentanil, cisatracurium, remifentanil, sevoflurane	Colon surgery	UL, 200mmHg, 5min × 3, before anesthesia induction	Uninflated cuff	MoCA
Zou 2013	China	R	68	49.7	53	Intravenous anesthesia with no volatile anesthetic agents	Cardiopulmonary bypass heart surgery	UL, 200mmHg, 5min × 3, after anesthesia induction and after CPB	Uninflated cuff	MMSE
Wang 2021	China	R	60	46	53.3	Etomidate, sufentanil, cisatracurium, remifentanil, desflurane	Moyamoya disease vascularization	LL, 200mmHg, 5min × 4, after anesthesia induction	Uninflated cuff	MoCA
Jing 2011	China	R, DB	40	49.5	37.5	Midazolam, etomidate, fentanyl, sevoflurane, Vecuronium bromide	On-pump valvular surgery	UL, 35kPa,5min× 4, after anesthesia induction	Uninflated cuff	MMSE and MoCA
Zhou 2018	China	R	60	69.5	56.6	Intravenous anesthesia with no volatile anesthetic agents	Liver cancer surgery	LL, 5min × 3, after anesthesia induction, arterial blood flow cannot be detected by ultrasound	Uninflated cuff	MMSE
Su 2009	China	R	36	55.5	41.6	Intravenous anesthesia with no volatile anesthetic agents	CPB heart surgery	UL, 200mmHg, 5min × 3, after anesthesia induction	Uninflated cuff	MMSE
Tao 2014	China	R	80	67.5	57.5	Intravenous anesthesia with no volatile anesthetic agents	radical resection of rectal carcmoma	LL, 200mmHg, 5min × 3, after anesthesia induction	Uninflated cuff	MMSE
Zhang 2020	China	R	69	67	49.2	Intravenous anesthesia with no volatile anesthetic agents	Surgical operation	UL, 50mmHg higher than basal systolic blood pressure (up to 200mmHg), 3min × 3, after anesthesia induction	Uninflated cuff	Z score

*RCTs* randomized controlled trials, *RIPC* remote ischemic preconditioning, *R* randomized, *DB* double-blind, *NR* not reported, *UL* upper limb, *LL* lower limb, *POCD* postoperative cognitive dysfunction, *SD* standard deviation, *MMSE* Mini-mental State Examination, *MoCA* Montreal Cognitive Assessment

### The effect of RIPC on the POCD incidence

**Fig. 3:**
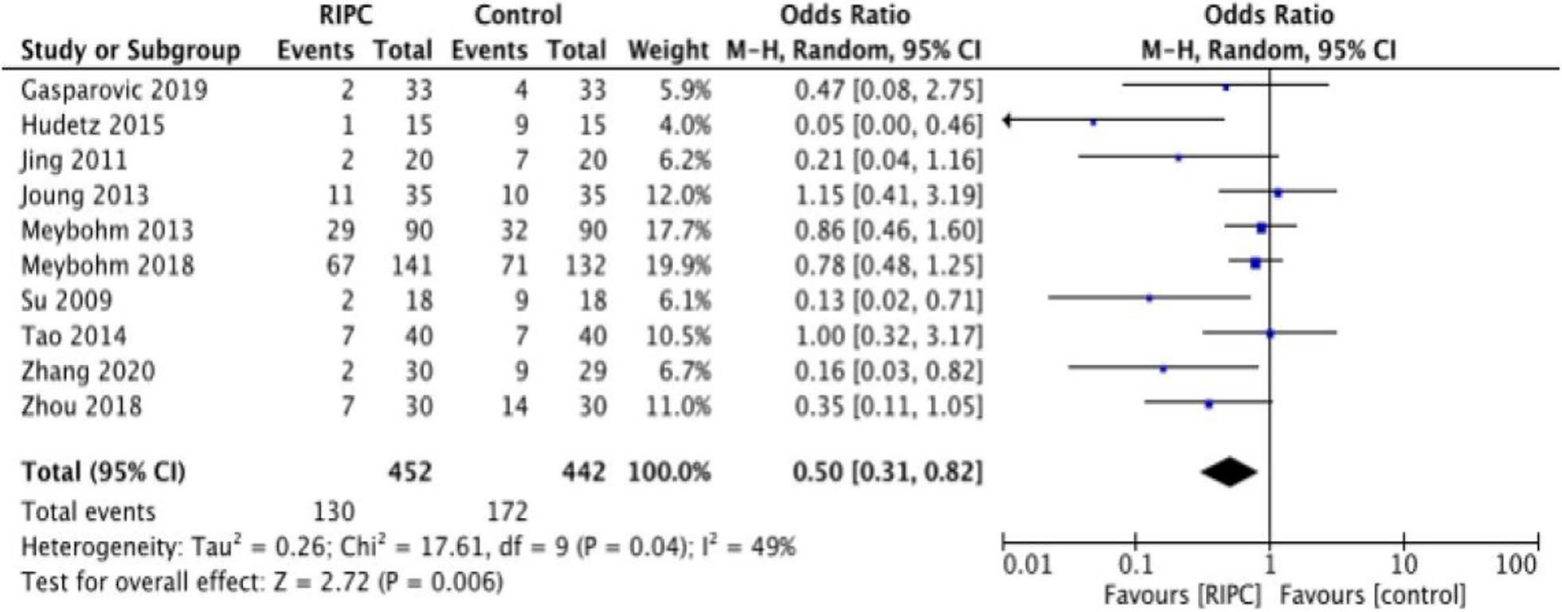
A forest plot for the meta-analysis of the effect of RIPC on POCD after general anesthesia surgery

Ten research with a total of 890 participants reported the efficacy of RIPC on the incidence of POCD 5–7 days after surgery. A random-effects model was chosen for meta-analysis in terms of moderate heterogeneity between trials (*P* = 0.04, *I*^2^ = 49%; Fig. 3). The outcome indicated that the incidence of POCD was significantly decreased in the RIPC group (OR = 0.50, 95% CI 0.31–0.82; Fig. 3). As shown in Table 2, the sensitivity analysis by excluding articles one by one had no significant effect on heterogeneity, indicating that our results were reliable. Meta-analysis of these ten articles proved that RIPC reduced the incidence of POCD 5–7 days after surgery.

**Table 2:** Sensitivity analyses

Study excluded	OR/MD (95% CI)	I^2^ (%)	P for Cochrane’s Q test	P for overall effect
*Influence of RIPC on POCD*				
Gasparovic 2019	0.49 [0.29,0.84]	54	0.03	0.009
Hudetz 2015	0.59 [0.38,0.91]	36	0.13	0.02
Jing 2011	0.53 [0.32,0.89]	50	0.04	0.02
Joung 2013	0.44 [0.26,0.76]	51	0.04	0.003
Meybohm 2013	0.43 [0.23,0.77]	51	0.04	0.005
Meybohm 2018	0.42 [0.23,0.79]	52	0.04	0.007
Su 2009	0.57 [0.35,0.91]	43	0.08	0.02
Tao 2014	0.45 [0.26,0.78]	53	0.03	0.004
Zhang 2020	0.56 [0.34,0.91]	46	0.07	0.02
Zhou 2018	0.52 [0.30,0.89]	51	0.04	0.02
*Influence of RIPC on surgery time*				
He 2017	−0.99 [−8.5,6.52]	54	0.04	0.8
Hudetz 2015	−2.43 [−7.68,2.82]	61	0.02	0.36
Jing 2011	−1.96 [−8.15,4.23]	60	0.02	0.53
Joung 2013	−3.18 [−8.18,1.82]	56	0.03	0.21
Wang 2021	−4.74 [−9.01, −0.47]	39	0.13	0.03
Zhang 2020	−2.79 [−7.95,2.38]	59	0.02	0.29
Zhou 2018	−1.8 [−5.09,1,49]	0	0.5	0.28
Zou 2013	−3.06 [−8.17,2.06]	57	0.03	0.24

*OR* odds ratio, *MD* mean difference, *CI* confidence interval, *POCD* postoperative cognitive dysfunction, *RIPC* remote ischemic preconditioning

### The association between RIPC and surgery time

**Fig. 4:**
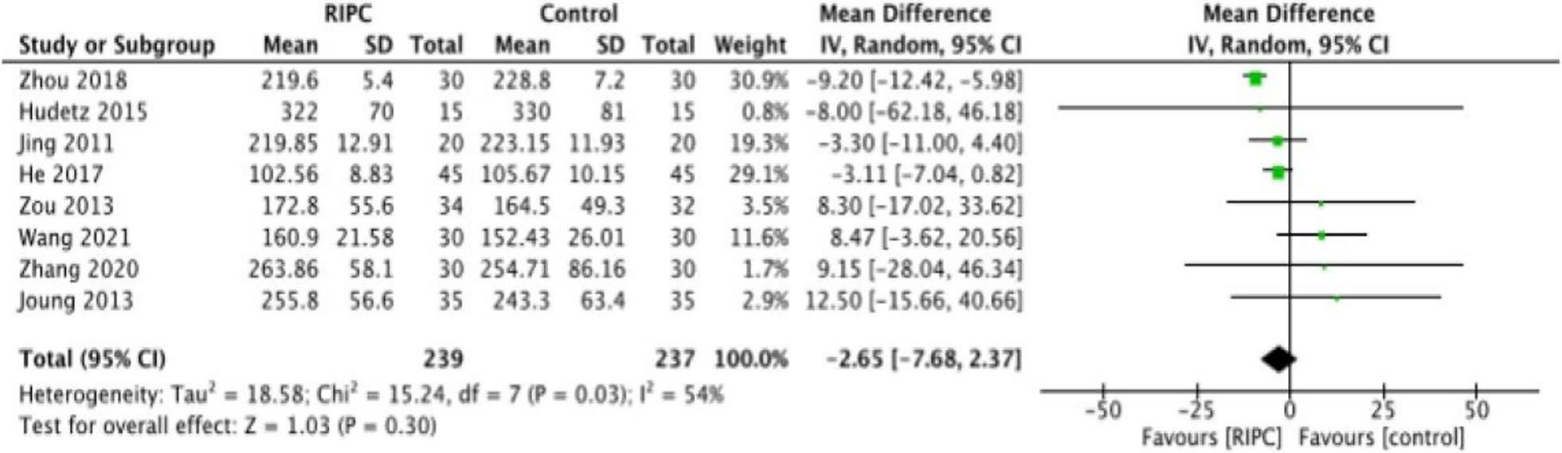
Mean differences for surgery time in patients between the RIPC group and the Control group (*P* = 0.30)

Eight articles involving 566 participants reported the operation time. Considering the apparent heterogeneity, the random-effects model was selected (*P* = 0.03, *I*^2^ = 54%; Fig. 4). The meta-analysis found that the surgery time was not extended in the RIPC group compared with the placebo group (MD = − 2.65, 95% CI − 7.68 to 2.37; Fig. 4). The results of the sensitivity analysis were shown in Table 1. There was moderate heterogeneity among the trials (*I*^2^ = 54%), the results must be interpreted cautiously. Notably, after excluding the Zhou 2018 study, the heterogeneity among RCTs was significantly reduced (*I*^2^ = 0%), indicating that this research was a primary source of heterogeneity.

### The association between RIPC and hospital stay

**Fig. 5:**
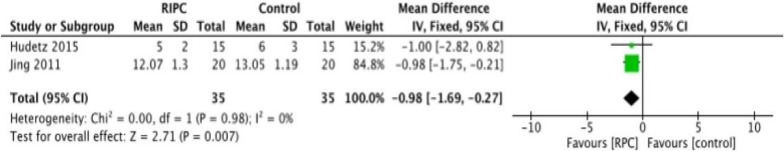
Mean differences for hospital stay time in patients between the RIPC group and the Control group (*P* = 0.007)

The efficacy of RIPC on the length of hospitalization was displayed in Fig. 5. Only two articles, including 70 patients, reported this result. Considering no heterogeneity between studies (*P* = 0.98, *I*^2^ = 0%), we used a fixed-effects model to analyze. Meta-analysis indicated that RIPC reduced the length of hospital stay (MD = − 0.98, 95% CI − 1.69 to − 0.27).

## Discussion

The meta-analysis included 13 randomized controlled trials with 1122 patients to assess the effectiveness of RIPC on POCD undergoing general anesthesia surgery in adults. The primary outcome proved that RIPC reduced the incidence of POCD in adult patients. The secondary result indicated that RIPC could shorten the length of hospital stay. As far as we know, this was the first meta-analysis to summarize the correlative literature on the impact of RIPC on POCD after general anesthesia surgery in adults. Based on the above results, RIPC could be used clinically to prevent POCD in adult patients under general anesthesia.

With the aging population trend in many countries, more and more elderly patients are receiving surgical treatment, and the incidence of POCD in older adults is expected to increase [31]. A recent systematic review suggested that age was the most common predictor of cognitive outcomes [32]. From the demographic characteristics in Table 1, the average age of patients was over 60 years in 8 of the 13 studies, partly explaining the higher incidence of POCD in the present meta-analysis. In addition, the duration of neuropsychological testing for included articles was 5–7 days after surgery. Previous studies suggested that POCD defined early after surgery might be a precursor to late complications [33]. Thus, the incidence of POCD within one week after the operation was a reminder of late prevention and was also of great significance for clinical practice guidelines.

In this meta-analysis, RIPC reduced the incidence of POCD in adult patients 5–7 days after surgery. The primary explanation might be that RIPC reduced the systemic inflammatory response and oxidative stress, including regulating inflammation-regulating genes, reducing neutrophil infiltration and inflammatory factors releasing [34, 35]. At the same time, the mechanism of POCD was associated with the neuro-inflammatory reaction [1]. Therefore, we hypothesized that the decreased systemic inflammatory response contributed to lower POCD incidence. However, insufficient data in the included literature prevented this meta-analysis from analyzing related inflammatory factors. The underlying mechanism needs to be discovered in future studies. In addition, the effectiveness of RIPC might be affected by the number and duration of ischemic cycles. However, a previous animal experiment has revealed that 4 and 6 cycles of ischemia/reperfusion in 5 min confirmed the same protective effect of RIPC [36], and this phenomenon was also proven in a clinical study [37]. Hence, the number of ischemic cycles was not the reason for the heterogeneity of the main results in this meta-analysis.

At present, there is no standardized neuropsychological test to accurately identify POCD [38]. For this reason, the literature included in this study contains different neurocognitive testing protocols, which might reduce the accuracy of the analysis. Differences in diagnostic tests between studies might also be one of the reasons for the heterogeneity of the primary outcome. Consequently, we performed a sensitivity analysis and found little change in heterogeneity after excluding one literature at a time, demonstrating that our conclusions were stable.

The higher POCD incidence and more severe symptoms might be due to the prolonged duration of surgery and the more complex type of surgery [39]. In our meta-analysis, RIPC did not prolong the operation time. Considering the heterogeneity of the results, we further performed a sensitivity analysis. It was noteworthy that heterogeneity between studies was reduced after excluding the study by Zhou 2018 (*I*^2^ from 54 to 0%), indicating that this article was a significant contributor to heterogeneity.

The advantages of this meta-analysis included rigorous literature screening and quality assessment. Moreover, sensitivity analysis was used to identify potential causes of heterogeneity. However, the limitations of this meta-analysis were as follows. First, publication bias could not be assessed because of the small number of eligible studies included. Second, the evaluation of neurological tests might have subjective reasons, which were easily influenced by individual factors. Finally, we should have performed the subgroup analysis on the type of operation, owing to the variety of surgical types and limitations in the number of studies. Therefore, the reliability of the trial needs to be improved in future studies by controlling for these two variables.

## Conclusion

In summary, this meta-analysis testified that RIPC could decrease the incidence of POCD in adult patients with general anesthesia and reduce hospital stay. The results of our meta-analysis might offer a new testimony to expand the clinical significance of RIPC.

## Data Availability

The datasets used and analyzed during the current study are available from the corresponding author on reasonable request.

## Abbreviations

POCD: Postoperative cognitive dysfunction
RIPC: Remote ischemic preconditioning
RCTs: Randomized controlled trials
MMSE: Mini-Mental State Examination
OR: Odds ratio
CI: Confidence interval
MD: Standardized Mean difference
R: Randomized
DB: Double-blind
NR: Not reported
UL: Upper limb
LL: Lower limb
SD: Standard deviation
MoCA: Montreal Cognitive Assessment
